# Mitochondrial medicine: “from bench to bedside” 3PM-guided concept

**DOI:** 10.1007/s13167-025-00409-4

**Published:** 2025-04-15

**Authors:** Qianwen Shao, Marie Louise Ndzie Noah, Olga Golubnitschaja, Xianquan Zhan

**Affiliations:** 1 Shandong Provincial Key Laboratory of Precision Oncology, Shandong Cancer Hospital and Institute, Shandong First Medical University and Shandong Academy of Medical Sciences, 440 Jiyan Road, Jinan, Shandong 250117 People’s Republic of China; 2 Predictive, Preventive and Personalised (3P) Medicine, University Hospital Bonn, Rheinische Friedrich-Wilhelms-University of Bonn, Venusberg Campus 1, 53127 Bonn, Germany; 3 Shandong Provincial Key Medical and Health Laboratory of Ovarian Cancer Multiomics, & Jinan Key Laboratory of Cancer Multiomics, Shandong Cancer Hospital and Institute, Shandong First Medical University and Shandong Academy of Medical Sciences, 440 Jiyan Road, Jinan, Shandong 250117 People’s Republic of China

**Keywords:** Predictive Preventive Personalized Medicine (PPPM / 3PM / 3P medicine), Mitochondrial medicine, Vital biosensor, Autophagy and mitophagy, Individualized patient profile, Health-to-disease transition, Metabolic disease, Cardio-vascular disease, Neurodegeneration, Cancer, Stress, Signaling, Environment, Chronic Fatigue, Cost-effective tailored treatments, Health policy

## Abstract

Mitochondria are the primary sites for aerobic respiration and play a vital role in maintaining physiologic function at the cellular and organismal levels. Physiologic mitochondrial homeostasis, functions, health, and any kind of mitochondrial impairments are associated with systemic effects that are linked to the human health and pathologies. Contextually, mitochondria are acting as a natural vital biosensor in humans controlling status of physical and mental health in a holistic manner. So far, no any disorder is known as happening to humans independently from a compromised mitochondrial health as the cause (primary mitochondrial dysfunction) or a target of collateral damage (secondary mitochondrial injury). This certainty makes mitochondrial medicine be the superior instrument to reach highly ambitious objectives of predictive, preventive, and personalized medicine (PPPM/3PM). 3PM effectively implements the paradigm change from the economically ineffective reactive medical services to a predictive approach, targeted prevention and treatments tailored to individualized patient profiles in primary (protection against health-to-disease transition) and secondary (protection against disease progression) healthcare. Mitochondrial DNA (mtDNA) properties differ significantly from those of nuclear DNA (nDNA). For example, mtDNA as the cell-free DNA molecule is much more stable compared to nDNA, which makes mtDNA be an attractive diagnostic target circulating in human body fluids such as blood and tear fluid. Further, genetic variations in mtDNA contribute to substantial individual differences in disease susceptibility and treatment response. To this end, the current gene editing technologies, such as clustered regularly interspaced short palindromic repeats (CRISPR)/Cas, are still immature in mtDNA modification, and cannot be effectively applied in clinical practice posing a challenge for mtDNA-based therapies. In contrast, comprehensive multiomics technologies offer new insights into mitochondrial homeostasis, health, and functions, which enables to develop more effective multi-level diagnostics and targeted treatment strategies. This review article highlights health- and disease-relevant mitochondrial particularities and assesses involvement of mitochondrial medicine into implementing the 3PM objectives. By discussing the interrelationship between 3PM and mitochondrial medicine, we aim to provide a foundation for advancing early and predictive diagnostics, cost-effective targeted prevention in primary and secondary care, and exemplify personalized treatments creating proof-of-concept approaches for 3PM-guided clinical applications.

## Preamble: mitochondria as a vital biosensor in human body

Mitochondria are the “powerhouse” of eukaryotic cells. Consequently, mitochondrial health and bio-energetic human health status are tightly linked together. Their indexation is used as objective metrics to assess mitochondrial functionality and bio-energetic efficacy status [1].

The mtDNA is lean on repair capacity, and physiologically predisposed to act as a potent genotoxic stress sentinel at the levels of cells and organisms [2]. Contextually, increased mitophagy (the key mechanism of mitochondrial homeostasis), which is reflected in systemic cell-free mtDNA circulation measurable in body fluids such as blood and tears [3–5], triggers protective systemic effects, which represents a physiologic stress response towards environmental changes, and redox imbalance. This signalling pathway is decisive to coordinate systemic processes such as cell proliferation, repair, and elimination, which makes mitochondria be the natural vital biosensor in humans.

However, persistent stress overload and extensive mitochondrial stress may result in mitochondrial burnout and sterile systemic inflammation, which collectively lead to irreversible mitochondrial injury and pathomechanisms to underlie downstream developing diseases [2, 6].

Mitochondrial burnout-associated pathologies can be exemplified by chronic fatigue, increased biologic vs. chronologic age known as a progressive aging, hormonal dysregulation and infertility, impaired healing, metabolic and mood disorders, autoimmune and eye disorders, severe respiratory diseases, neurodegeneration and malignant cell transformation [2].

Further, an increased mitophagy acts as a direct activator of the nDNA repair machinery under genotoxic conditions that has to be carefully considered when treating severe pathologies such as anti-cancer therapies, in order to avoid chemoresistance of cancer tissue [7]. Therefore, the targeted manipulation of mitochondrial homeostasis is a promising strategy in innovative treatment algorithms tailored to individualized patient profiles [8].

Finally, to adapt to the ever-changing environment, mitochondria are a highly dynamic subcellular population. Decreased mitochondrial dynamics is associated with impaired mitochondrial homeostasis and severe systemic disorders [9] which is an attractive target for 3PM-guided mitochondrial medicine [2].

## Attributes of mitochondrial health and dynamics

### Basic information

In the earth history, about two billion years ago the archaea, instead of to combat all the invaded bacteria, started to tolerate some of them. This was such a learning process to adapt the “newcomers” by subordinating them and taking great advantages of this collaboration such as an effective production of the energy resources and heating. Coming together into business stepwise turned into the unique form of the life-important “partnership” created between human cells and their essential subcellular units such as mitochondria [7].

Mitochondria are composed of mitochondrial matrix, inner mitochondrial membrane (IMM), mitochondrial membrane gap, and outer mitochondrial membrane (OMM). Mitochondria are center of aerobic respiration, producing about 95% of the total energy required for all cellular activities. Mitochondrial proteins are encoded by mitochondrial DNA (mtDNA) and neuclear DNA (nDNA). Of them, mtDNA is self-replicating and transmits genetic information, which contains 37 genes encoding 14 proteins [10], assembled within mitochondrial complexes I, III, IV, and V [11–13]. Complex I includes 7 subunits (nicotinamide adenine dinucleotide (NADH)-ubiquinone oxidoreductase chain 1 (ND5), ND1, ND4, ND2, ND3, ND4L, and ND6), complex III includes 1 subunit (cytochrome b; cytb), complex IV includes 3 subunits (cytochrome c oxidase subunit 1 (COXI), COXII, and COXIII), and complex V includes 2 subunits (ATP synthases 6 and 8). The most of mitochondrial proteins are encoded by nDNA. The nDNA-encoded protein precursors are initially synthesized in ribosome in the cytoplasm, then these synthesized protein precursors are transported to mitochondria. To smoothly introduce these precursor proteins into mitochondria, cells rely on a series of specific protein complexes as follows. First, the outer membrane transferase (TOM) complex assists precursor proteins in crossing the OMM. Second, the TIM23 complex promotes their further crossing of the mitochondrial inner membrane. Third, the sorting and assembly machinery (SAM) complex is responsible for the appropriate protein locations to ensure their functionality [14]. The TOM complex, which consists of TOM22, TOM20, TOM70, TOM4, TOM5, TOM6, and TOM40, is responsible for the protein entry into the membrane gap through OMM. The SAM complex in OMM contains three subunits, including SAM50, SAM35, and SAM37, and can mediate the assembly of TOM core complexes [14]. The TIM23 complex is the main transportation and assembly complex of IMM, containing at least 11 proteins, such as TIM23, TIM50, TIM17, TIM21, PAM17 (presequence translocated-associated motor subunit PAM17, mitochondrial), PAM18 (mitochondrial import inner membrane translocase subunit TIM14), MGR2 (mitochondrial genome-required protein 2), and mtHSP70 (mitochondrial heat-shock protein 70) [15].

Mitochondrial precursor proteins are directed by two types of targeting signals—cleavable precursor sequences and non-cleavable multiple integrated localization signals, and the most classic transport pathway is the sequence pathway [16]. Proteins are transported to the mitochondrial matrix through peptide transport pathways, and the localization and sorting signals of proteins are cleavable leading sequences that are transported by the TOM complex in the OMM [17–19]. The precursor protein is in an extended state when transported to the mitochondrial matrix, and the cleavage sites are recognized as mitochondrial processing peptidases (MPP). The precursor protein first binds to the outer membrane surface receptor TOM20 and is then transferred to TOM22. With the assistance of TOM5, the precursor protein passes through transmembrane TOM-complex channel in an unfolded state and passes through the N-terminus first. Under the synergistic effect of two subunits TIM50 and TIM23 in the TIM23 complex, it was pulled onto the surface of the TIM23 complex. Driven by IMM potential and energy provided by the hydrolysis of ATP by mtHSP70, the precursor protein crosses the TIM23 transmembrane channel and sequentially binds to TIM44 and mtHSP70, ultimately being pulled into the mitochondrial matrix by mtHSP70. Finally, in the matrix, MPP hydrolyzes the precursor protein to cleave the lead peptide and fold to form a mature protein.

### Mitochondrial quality control and systemic effects by compromised mitochondrial health

**Fig. 1:**
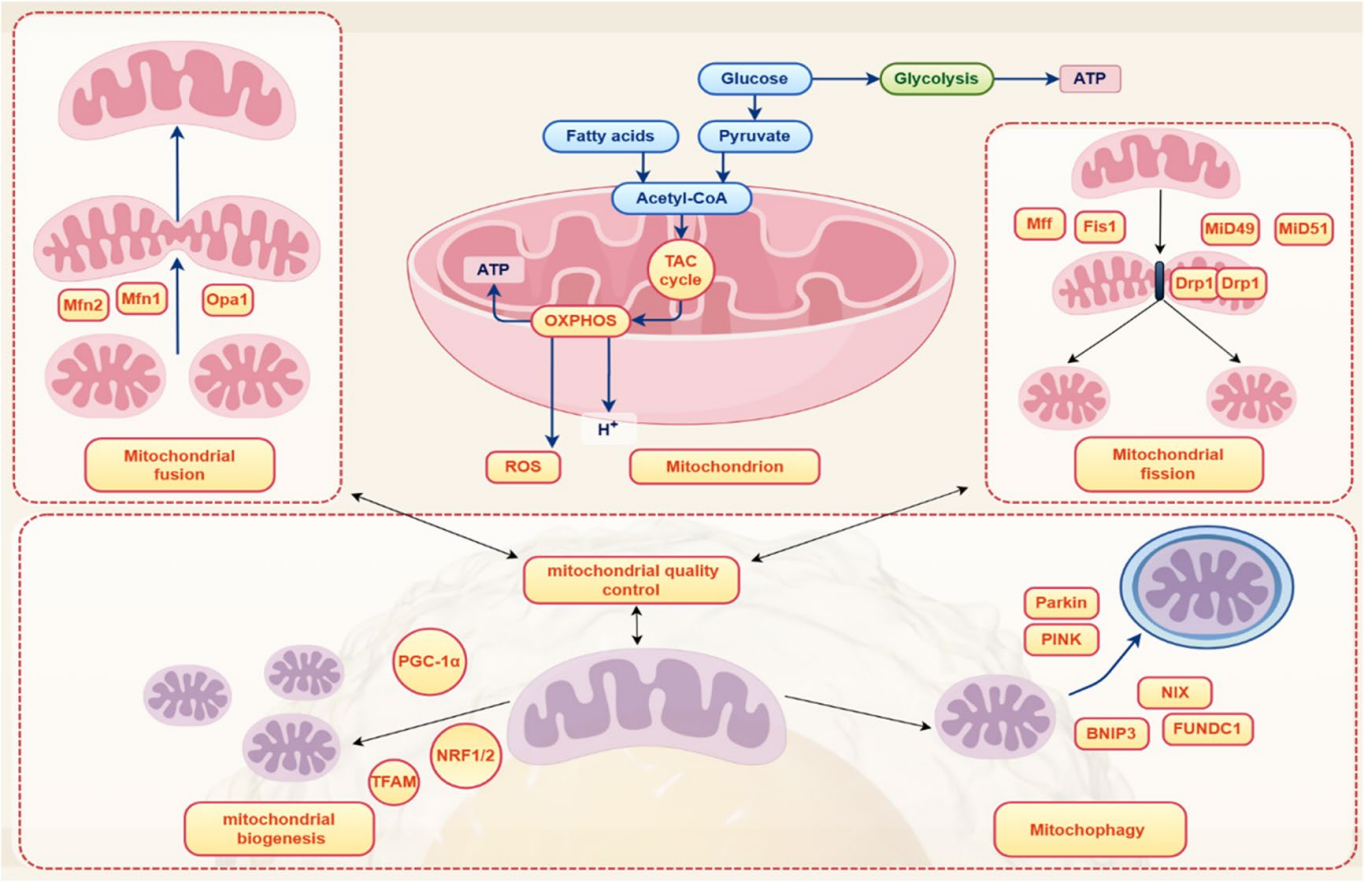
Mitochondrial quality control is based on regulatory processes of biogenesis, homeostasis, dynamics, and autophagy; mitochondrial dynamics is controlled by mitochondrial fusion proteins Mfn1/2 and Opa1, and mitochondrial fission proteins Drp1 and Fis1; mitochondrial biogenesis is controlled by PGC- 1α, TFAM, and NRF1/2; and mitochondrial autophagy (mitophagy) is controlled by PINK/parkin proteins

Mitochondria adapt to the needs of cells through mitochondrial dynamics and homeostasis [9] as highlighted in Fig. 1. Mitochondrial fusion is controlled by proteins Mfn1 (mitofusin- 1); opa1 (optic atrophy 1, which is a mitochondrial dynamin-like GTPase); and Mfn2 (mitofusin- 2). Opa1 drives the IMM fusion, and Mfn1 and Mfn2 drive the OMM fusion [20, 21]. Mitochondrial fission is regulated by Fis1 (mitochondrial fission protein 1) and Drp1 (dynamin-related protein 1) [22]. Drp1 receptors (Mid51 (mitochondrial dynamics protein MIEF1), Mid49, Mff (mitochondrial fission factor), and Fis1 (mitochondrial fission 1 protein)) exist in the OMM. The process of mitochondrial division first involves phosphorylation of Drp1, and then recruited to the OMM by interacting with its receptors, Drp1 is then assembled into a circular structure, surrounding and compressing mitochondria, consuming guanosine triphosphate (GTP), and producing two separate mitochondria [22].

Impaired mitochondrial homeostasis (mitophagy, fusion and fission) disrupts the mitochondrial dynamics balance resulting, e.g., in excessive fusion or insufficient fission, mitochondrial stress or even mitochondrial burnout—all reflected in abnormal mitophagy and disrupted metabolic and key signaling pathways at the levels of cells and organisms [1]. This results in excessive reactive oxygen species (ROS) production, changes in energy metabolism, dysregulation of calcium homeostasis, and alterations in mitochondrial enzyme activity. Mitochondrial fusion proteins can regulate apoptosis by inhibiting cytochrome C, and autosomal dominant optical atrophy (ADOA) is caused by opa1 mutations. Abnormal mitochondrial fusion and fission are involved into the central mechanisms of cancer development. Mitochondrial elongation factor 2 (MIEF2) is involved in mitochondrial fission, and its expression is increased in ovarian cancer, which enhances the Warburg effect in ovarian cancer cells, and promotes oxidative phosphorylation (OXPHOS) towards aerobic glycolysis, and it is crucial to ovarian cancer cell’s migration and invasion [23]. MARCH5 (membrane-associated RING-CH 5) affects mitochondrial fission and is highly expressed in epithelial ovarian cancers. Increased MARCH5 expression makes it be a potential biomarker in ovarian cancer, because it can promote the migration and invasion in ovarian cancer [24]. Loss of Mfn1 function triggers epithelial mesenchymal transition in hepatocellular carcinoma (HCC), which is supportive for HCC invasiveness and metastasis. Drp1 is increased in invasive breast cancer and lymph node metastasis. Further, silent Drp1 and overexpressed Mfn1—both suppress breast cancer metastases; and cancer cell migration and invasion can be enhanced by inhibiting mitochondrial fusion [25].

Mitochondrial quality is controlled by several alternative mechanisms of mitochondrial autophagy (usually linked to apoptosis) and mitophagy (targeted elimination of damaged mitochondria from the cell). Mitophagy is a selective autophagy that transports mitochondria to lysosomes for clearing excessive, aged and damaged mitochondria. Well-known mitophagy triggers are excessive ROS production, protein misfolding, and mitochondrial depolarization. Depolarized mitochondria are enveloped by autophagosomes, and afterwards fused with lysosomes to degrade “labeled” mitochondria. There are two main pathways for mitophagy. (i) The first pathway is the ubiquitin-dependent pathway mediated by PINK1 and parkin [22, 26]. PINK1 interacts with parkin to control mitophagy and maintain high mitochondrial quality. PINK1 is blocked from entering IMM and accumulated on the OMM when mitochondrial membrane potential (MMP) is damaged, which leads to its phosphorylation. Parkin is activated to convert PINK1 into activated E3 ubiquitin ligase, which can ubiquitinate OM protein and attract the adaptor sequestosome- 1 (SQSTM1/p62) to ubiquitin chain, then microtubule-associated protein 1 light chain 3 (LC3) binds to P62 and ultimately completes mitophagy. Study has shown that SQSTM1/p62 can promote mitochondrial ubiquitination, but its function is independent of PINK1 and parkin during mitophagy [27]. (ii) The second pathway is a non-ubiquitin-dependent pathway, which is mediated by receptors [22]. Autophagy receptors (such as NIX (Nip3 like protein X), BNIP3 (BCL2/adenovirus E1B 19 kDa protein-interacting protein 3), and FUNDC1 (FUN14 domain containing 1)) are recruited by PINK1 through ubiquitin phosphorylation, including FUNDC1 receptor, Bcl2-(B-cell lymphoma 2)-interacting protein 3 (BNIP3) receptors, and NIX receptors, the receptor proteins recruit LC3, which enables autophagosomes to engulf mitochondria [22].

Physiologic biogenesis of mitochondria governs their quality control and maintains mitochondrial health and cell survival. Peroxisome proliferator-activated receptor (PPAR)-γ coactivator- 1α (PGC- 1α) is considered to regulate mitochondrial production. Sestrin2 (Sesn2), as a novel stress-induced protein, has been shown to reduce ROS production. Overexpression of Sesn2 can alleviate osteoarthritis pain by inducing AMP-activated protein kinase (AMPK)/PGC- 1α mediated mitochondrial biogenesis [28]. The silent information regulator 3 (SIRT3)/PGC- 1 α pathway, regulated by α-arbutin, can improve UVA-induced photoaging. In this pathway, SIRT3, PGC- 1α, and MMP are significantly increased due to the regulation of α-arbutin. α-arbutin can also mediate mitochondrial biogenesis and initiate downstream signaling, thus significantly improve the structure of mitochondria [29]. When oxygen conditions are normal, lysine (K) demethylase 3 A (KDM3 A) can bind to PGC- 1α and demethylate monomethylated lysine (K) 224 of PGC- 1α. In a hypoxic environment, KDM3 A is stimulated, PGC- 1α K224 monomethylation can be enhanced, which decreases PGC- 1α’s activity required for nuclear respiratory factor 1 (NRF1)- and nuclear respiratory factor 2 (NRF2)-dependent transcriptional regulations of TFB1M (dimethyladenosine transferase 1, mitochondrial); TFB2M (dimethyladenosine transferase 2, mitochondrial); and TFAM (transcription factor A, mitochondrial), to result in reduced mitochondrial biogenesis [30].

## Mitochondrial health status-driven diseases

### Cardiovascular diseases

Heart failure, cardiac ischemia–reperfusion injury (IRI), hypertrophic cardiomyopathy (HCM), and coronary artery disease have been shown that their pathogenesis is related to mitochondria. Human heart relies heavily on the energy produced by mitochondria to maintain its basic function, and approximately 95% of the ATP consumed by the heart comes from mitochondria [31]. The energy stored in the heart can only sustain a heartbeat for a few seconds [31]. In the heart, mitochondria maintain the most basic energy supply by consuming fatty acids via oxidative metabolism. During the pathological state, mitochondrial reprogramming of energy metabolism can lead to insufficient energy supply, resulting in heart failure [32]. Mitochondrial proteins that are highly acetylated are related to heart failure [32]. Elevated mitochondrial acetylation, including proteins involved in substrate oxidation such as pyruvate dehydrogenase and fatty acid oxidase, has been found in animal models and heart failure patients. These proteins have been shown to exhibit high levels of acetylation [33–37]. In addition, the substrate metabolism and OXPHOS have a negative feedback on mitochondrial acetylation, which makes a further impair on energy metabolism. Furthermore, a significant mechanism contributing to the increase in protein acetylation is the reduction in protein deacetylation, which is regulated by sirtuin family nicotinamide adenine dinucleotide (NAD)-dependent deacetylases. The most important mitochondrial deacetylase in the sirtuin family is SIRT3, which is reduced in myocardium with heart failure [37]. Acetylated proteomic analysis showed the specific lysine acetylation, and the overall acetylation of two central mitochondrial metabolic enzymes (pyruvate dehydrogenase (PDH) and ATP synthase) increased. The use of SIRT inhibitors or SIRT3 knockdown enhances their acetylation levels to ultimately affect mitochondrial functions [37].

Mitochondrial dysfunction may lead to increased oxidative stress, trigger inflammatory reactions, result in insufficient energy supply to myocardial cells, exacerbate myocardial ischemia and reperfusion injury, and ultimately lead to cardiovascular disease. The molecular mechanism of cardiac IRI involves multiple cellular components. Cardiac ischemia causes a decrease in OXPHOS, loss of mitochondrial membrane depolarization, accumulation of lactate, and a decrease in intracellular pH. The accumulation of H^+^ in the cell activates Na^+^/H^+^ and inhibits Na^+^/K^+^ ATPase. Subsequently, the Na^+^/Ca^2+^ exchange function is reversed, leading to cytoplasmic Ca^2+^ overload. After reperfusion to restore oxygen transportion, Ca^2+^ overload and unregulated ROS can lead to MPTP (mitochondrial-permeability transition pore) and a non-specific inner membrane channel opening. MPTP opening leads to mitochondrial membrane permeabilization, which causes depolarization, swelling, and rupture of mitochondria, and results in myocardial cell damage. Therefore, MPTP has always been a therapeutic target for preventing IRI [38]. In addition, mitochondrial transplantation can improve heart disease by directly and safely delivering mitochondria to the heart through injection and intravascular delivery, thereby improving IRI [39]. Clinical trials have shown that patients undergoing mitochondrial transplantation have better postoperative recovery ability.

### Mitochondrial diabetes mellitus

Mitochondrial diabetes mellitus (MDM) accounts about 3% in total number of diabetes, which is a special type of diabetes caused by mitochondrial enzyme deficiency. MDM differs from the other subtypes of diabetes during clinical treatment. Therefore, early and personalized diagnosis and treatment will help patients with follow-up treatment. Abnormal mitochondrial function may cause insulin resistance and insufficient insulin secretion, which leads to an increase in blood sugar, thus causing diabetes. The OXPHOS in MDM patients was found to be reduced, and mitochondrial dysfunction could lead to insulin resistance, resulting in diabetes [40, 41]. Genomic analysis of human diabetic muscle shows downregulated the expresson of PGC- 1α expression that is related to OXPHOS [42]. SIRT1 is involved in numerous physiological processes such as DNA damage repair, gene transcription, energy metabolism, stress, and apoptosis. Under nutritional limitations, energy homeostasis is maintained by increasing mitochondrial fatty acid oxidation in peripheral tissues, and SIRT1 can regulate PGC- 1α metabolic adaptation based on acetylation levels [43]. As an activator of SIRT1, resveratrol induces PGC- 1α’s activity to improve insulin sensitivity in high-fat diet obese mice and protects them from the development of obesity and insulin resistance [44].

Mitochondrial dysfunction causes many complications, such as diabetes kidney disease (DKD) and diabetes with nonalcoholic fatty liver disease, diabetes cardiomyopathy, and diabetes ulcer. DKD is the most common microvascular complication in diabetes patients, usually accompanied by a large amount of proteinuria. The damage to podocytes is related to the occurrence of DKD and mitochondrial abnormalities [45]. AKAP1 (A-kinase anchor protein 1, mitochondrial) activates phosphorylated La-related protein 1 (Larp1) through PKC signaling to reduce mtDNA replication, thereby accelerating mitochondrial dysfunction and podocyte damage in DKD. Type 2 diabetes (T2D) can destroy mitochondrial integrity, release mitochondrial ROS and mtDNA into the cytoplasm, and activate NLRP3 (NOD-like receptor with pyrin domain 3) inflammasome. NLRP3 inflammasome is associated with vascular complications in T2D. NLRP3 inflammasome inhibitors such as daggligin can alleviate cardiomyopathy in T2D by inhibiting NLRP3 inflammasome activation. Shagliptin can also inhibit the activation of NLRP3 in kidney and adipose tissue in T2D and can improve the process of myocardial lesion in diabetes when combined with epallegin. Rhodiola glycoside can pass through the SIRT1-PGC- 1α ways to improve DKD [46]. As an intervention drug for diabetes treatment, metformin can reduce ROS production, activate AMPK/PGC- 1/NRFs axis, increase mitochondrial biogenesis, and improve mitochondrial function. Thiazolidinediones and angiotensin-converting enzyme inhibitors can also improve mitochondrial function and increase its biogenesis [47]. Studies on clinical outpatient populations have shown that the expression of mitotic related proteins is high in T2D patients but related fusion proteins is low, and the use of metformin in patients can improve mitochondrial dynamics [48].

### Neurological and neurodegenerative diseases

Mitochondrial damage can cause neurological diseases, such as Leber’s hereditary optic neuropathy, Alzheimer’s disease (AD), Huntington’s disease, and Parkinson’s disease (PD). Mitochondrial defects or dysfunction have become one of its new pathologies [49, 50]. AD is a common neurodegenerative disease in humans; in AD, the bioenergy function of mitochondria is reduced, which manifests that the enzyme activity in the Krebs cycle, respiratory chain activity, OXPHOS, and ATP production are decreased, and ROS is increased [49, 51].

The pathology of AD is related to calcium homeostasis. With the increase of human’s age, the regulatory function of mitochondria on calcium is weakened, and amyloid beta peptide (Aβ) causes calcium ions to aggregate, which leads to excessive calcium is uptake by mitochondrion, thus affects mitochondrial functions and increases free radicals [52]. In AD, tubulin associated unit (Tau) protein undergoes abnormal post-translational modifications (PTMs) such as phosphorylation, leading to the formation of NFT (neurofibrillary tangle), which is a significant pathological feature of AD.

In AD cells, mitochondrial axonal function and mitochondrial dynamics are disrupted, and organelles are more inclined towards perinuclear distribution. Tau is related to mitochondrial dysfunctio because it can stabilize actin and inhibit Drp1, leading to mitochondrial elongation. Excessive mitochondrial fission can lead to cognitive decline. Drp1 interacts with Aβ, and partial defects in Drp1 can lead to Aβ reducing. The use of Drp1 inhibitors such as p110 and Mdivi- 1 (mitochondrial Division Inhibitor 1) can improve learning and cognitive abilities. Mitochondria-associated endoplasmic reticulum membrane (MAM) connects mitochondria and endoplasmic reticulum to mediate their signal communication, participate in cholesterol synthesis metabolism, and regulate calcium homeostasis. AD has experienced many mitochondria-related metabolic disorders, and C99 (the amyloid precursor protein 99-aa C-terminal fragment) in MAMs is the direct precursor of Aβ. The increase of C99 in AD leads to mitochondrial dysfunction [51]. The quantitative proteomics analysis of MAM isolated from 3-month-old amyloid precursor protein/presenilin 1 (APP/PS1) mice found that Aβ overexpression is associated with changes in the MAM proteome of the mouse cerebral cortex [53]. Among 5957 proteins identified in mouse MAM, 128 proteins are localized in mitochondria, endoplasmic reticulum, or ribosome, and most of them are involved in protein synthesis, folding, and degradation. HSPA1b is over-expressed in the APP/PS1 mice, which is a chaperone protein that is important in the aggregation of toxic misfolded proteins. Also, oxidative stress response-related proteins and endoplasmic reticulum-related protein degradation are increased in the APP/PS1 mice [53].

SIRT1 protein is associated with neuroprotection. SIRT1 upregulates PGC- 1 α and activates major autophagy proteins (ATG5, ATG7, and ATG8/LC3) through deacetylation and stabilization of PINK1, as well as upregulation of mitochondrial autophagy receptors Nix/BNIP3L and LC3. Therefore, targeting SIRT1-PGC- 1α pathways may improve mitochondrial damage and neurological diseases [54, 55]. The function of cyclin dependent kinase 5 (CDK5) in the nervous system plays important roles in regulation of mitochondrial division and neuronal apoptosis processes. This mechanism makes CDK5 be a potential therapeutic target, which is expected to provide new ideas to treat neurodegenerative diseases [56]. PGC- 1α is an important regulatory factor in the transcriptional regulation of β-APP cleaving enzyme 1 (BACE1) in the pathogenesis of AD. BACE1 is an enzyme that promotes the production of Aβ, and its expression level is reduced in AD patients, further exacerbating the progression of this disease.

Autopsy analysis of post-mortem AD brain tissues found that mitochondrial biosynthesis-related genes were reduced. Among them, the expressions of genes such as PGC- 1α, TFAM, and NRF2 were inhibited in AD patients, which provide important clues to a deeper understanding of AD mechanism [57]. Upregulation of PGC- 1α can inhibit the progression of AD, suggesting that this pathway might be a new strategy to treat AD in the future. Therefore, exploration of the relationship between CDK5 and PGC- 1 α might be a new direction to develop new therapies and interventions.

PD is a genetically influenced, common neurodegenerative disease, so fair more than 100 genes or genetic loci have been identified [58]. Among the functional enzymes of mitochondria, the enzyme NDUFS2 in complex I affects the progression of PD. NDUFS2 is the key structure of complex I in mouse dopaminergic neurons, its knocking out in the mice exhibited mitochondrial dysfunction and neurodegenerative symptoms [59]. Previous studies have confirmed that the absence of IFNβ or IFNAR1 (the receptor for IFNα/β) can lead to pathological and behavioral changes similar to PD. Transcriptomic analysis found an increase in sporadic PD (sPD)-related PIAS2 (protein inhibitor of activated STAT 2). In addition, sPD patients express higher levels of PIAS2 mRNA and protein in neurons. Overexpression of PIAS2 in mice has been confirmed to indeed lead to motor and cognitive impairments [60]. For a long time, the etiology of PD is closed with mitochondrial dysfunction. PINK1 is a mitochondrial membrane protein, which maintains mitochondrial autophagy and protects nerve cells. PINK1 is imported to arrest on the translocase of TOM complex, which activates its autophosphorylation-dependent ubiquitin kinase activities, and initiates Parkin-dependent mitochondrial clearance. To cope with mitochondrial stress, PINK1 is accumulated and bond to the core components of TOM and TIM23 complex, resulting in assembly of 720 kDa PINK1-TOM-TIM23 super complex [61, 62].

### Cancers

Compared to normal cell that relies on OXPHOS for energy, tumor cell relies on aerobic glycolysis for energy, which called Warburg effect. The mitochondrial function of tumor cells often undergoes abnormalities, including increased lactate production gluconeogenesis, glutamic acid hydrolysis activity, and decreased fatty acid oxidation and pyruvate oxidation. The activities of certain mitochondrial enzymes that are essential for OXPHOS are decreased in cancer relative to normal cells, including adenine nucleotide translocatase, ATPase, and cytochrome C oxidase.

For example, ovarian cancer is considered a serious gynecological tumor with a high mortality rate [63]. GLOBOCAN data show that there were 324,398 newly diagnosed cases of ovarian cancer worldwide in 2022, resulting in 206,839 deaths of ovarian cancer patients worldwide [64]. Ovarian cancer has a strong early concealment, and most patients are diagnosed in the late stage. Under a microscope, the mitochondria of ovarian cancer tissue were swelling with reduced mitochondrial cristae, and significant changes in morphology and structure compared to normal ovarian cancer tissue [65]. Furthermore, proteins participate in the execution of biological functions and may be modified in various ways after translation, such as phosphorylation, acetylation, ubiquitination, and methylation, which are important way to reveal mitochondrial and cancer-related diseases, and contribute to more precise cancer research in the context of predictive, preventive and personalized medicine (PPPM; 3PM). The isobaric tags for relative and absolute quantitation (iTRAQ) quantitative proteomics revealed 1198 mitochondrial differentially expressed proteins (mtDEPs) [65] and mitochondrial phosphoprotein profile [66] in human ovarian cancer mitochondria, which provided new insights into the roles of mitochondria in ovarian cancer, and found metabolic pathways related to energy metabolism reprogramming in ovarian cancer [66].

Mitochondria mediate energy metabolism reprogramming, regulating nutrient metabolism and processes such as cancer occurrence, drug resistance, and invasion. During the development of cancer, the tumor microenvironment (TME) is associated with energy reprogramming. The TME has immunosuppressive properties, which prevents the immune system from detecting and eradicating malignant cells [67]. In TME, immune cells, cancer cells, and stromal cells compete for nutrients, this competition helps tumor cells evade immune surveillance and promotes the growth of tumor cell [68].

The epigenetic silencing of Otubain 2 (OTUB2) promotes mitochondrial metabolic reprogramming and drug resistance in ovarian cancer. The silence of OTUB2 disrupts the stability of the sorting nexin 29 pseudogene 2 (SNX29P2) pseudogene, thereby preventing the elevation of hypoxia inducible factor- 1α (HIF- 1α) and activating the transcription of carbonic anhydrase 9 (CA9) [69]. Glutamine metabolism, a nutrient is widely involved in proliferation, invasion, metastasis, drug resistance, and immune escape in ovarian cancer, which makes it be a potential therapeutic target [70–72]. Tumor cell has a rapid proliferation, de novo synthesis of glutamine cannot meet the needs of tumor cell, glutamine can convert into a conditionally essential amino acid, which promotes malignant progression of tumors. Glutamine is crucial in regulating immune cell activity, and high levels of extracellular glutamine are essential for the production and phagocytosis of cytokines in macrophages. Cancer cells use a large amount of glutamine to generate energy through the TCA cycle, inducing glutamine depletion in the TME to affect the function of immune cells. Fatty acids released by tumor cells promote tumor growth by affecting various immune cells in the TME, including natural killer cells, dendritic cells, T cells and neutrophils. Abnormal lipid metabolism is also an important metabolic change in ovarian cancer, such as changes in cholesterol, glycerophospholipids, sphingophospholipids, and their related metabolic pathways, which lead to drug resistance in ovarian cancer, thereby affecting the progression of tumor [73–75]. Clinical studies have detected the accumulation of important lipids and intermediate metabolites in the ascites, plasma, or ovarian cancer cells of patients with malignant ascites and ovarian cancer. The expressions of key proteins and related enzymes, which participate in lipid synthesis and breakdown pathways, are significantly upregulated, and the accumulation of related metabolites is associated with tumor differentiation and prognosis. In high-grade serous ovarian cancer (HGSOC), the accumulation of lipid droplets (LD) is closely associated with survival-related pathways and poor prognosis. Patients with HGSOC with more lipid droplets LD in their cells have lower survival rates [76]. In ovarian cancer, drug resistance is a very common issue. Tissue analysis of ovarian cancer patients confirmed that acylglycerol kinase promotes chemotherapy resistance in epithelial ovarian cancer through interaction with ribosomal protein L39 [77]. Mitochondrial dysregulation is closely related to diseases such as ovarian cancer, CRL4^CUL4 A/DDB1^ regulates chemotherapy resistance in ovarian cancer cells by modulating mitochondrial dynamics and autophagy. CRL4^CUL4 A/DDB1^ enhances mitochondrial division by upregulating AMPKα^Thr172^ and MFF^Ser172/Ser146^ phosphorylation, thereby recruiting Drp1 to mitochondria. The deletion of CRL4^CUL4 A/DDB1^ stimulates mitophagy through PINK1-Parkin pathway to degrade mitochondria. In addition, the absence of CRL4^CUL4 A/DDB1^ inhibits the proliferation of ovarian cancer cells, while inhibition of autophagy partially reverses this disruption [78].

Mitochondrion also works in the development of other cancers. Cell apoptosis that is regulated by mitochondrion affects lung cancer. Studies have shown that apoptosis-inducing factor (AIF) regulates mitochondrial respiration and OXPHOS. In the absence of AIF, OXPHOS and mitochondrial structure can be damaged, which enhances glycolysis and sensitivity to glucose deprivation [79]. A conserved 90-amino acid peptide encoded by lncRNA AFAP1-AS1 (actin filament associated protein 1 antisense RNA 1) is located on mitochondria, which is called as an AFAP1-AS1 translated mitochondrial‐localized peptide (ATMLP), promoting the malignant phenotype of non-small cell lung cancer. Our team identified the relevant signaling pathways of pituitary tumor mitochondria, including glycolysis and oxidative stress, through proteomic techniques, and found that the pathogenesis of pituitary tumors is closely related to mitochondrial dysfunction [80, 81]. Mitochondrial uncoupling protein 2 (UCP2) is a potential target for cancer drug therapy in pancreatic cancer [82, 83]. UCP2 is necessary for Drp1 mediated mitochondrial fission, and UCP2 deficiency leads to loss of Drp1 phosphorylation. The reduction of mitochondrial localization in triple negative breast cancer (TNBC) leads to a large accumulation of PD-L1, which is not conducive to the combined immunotherapy of immune checkpoint inhibitor (ICI) and paclitaxel. ATAD3 A protein encoded by nuclear gene can regulate mitochondrial dynamics and affect the progress of tumor. Targeting ATAD3 A protein is expected to improve the ability of immunotherapy [84]. Most patients with advanced TNBC will develop drug resistance [85]. After neoadjuvant chemotherapy, Myc proto-oncogene protein (MYC) and myeloid cell leukemia- 1 (MCL1) undergo frequent co amplification in drug-resistant TNBC, increasing OXPHOS and ROS production, thereby maintaining drug resistance.

Pathologies considered to be directly linked to compromised mitochondrial health are exemplified in Table 1.

**Table 1:** Prominent examples of pathologies directly linked to compromised mitochondrial health. *MnSOD*, Mn-superoxide dismutase; *FARS2*, phenylalanine-tRNA ligase mitochondrial; *HUVECs*, human umbilical vein endothelial cells; *NDUFB5*, NADH-ubiquinone oxidoreductase subunit B5; *MELAS*, mitochondrial encephalomyopathy, lactic acidosis, and stroke-like episodes; *OTUB2*, otubain 2; *AIF*, apoptosis-inducing factor; *ATMLP*, AFAP1-AS1 translated mitochondrial-localized peptide; *MARS2*, mitochondrial methionyl-tRNA synthetase; *MCU*, mitochondrial calcium uniporter, *NOX*, NADPH oxidases; *Prx- 3*, peroxiredoxin- 3; *AGO2*, argonaute 2; *Bmal1*, brain and muscle arnt-like protein 1; *IP3R*, inositol 1,4,5-trisphosphate receptor; *METTL3*, methyltransferase-like 3; *HSF1*, heat shock transcription factor 1; *NDUFS2*, NADH dehydrogenase [ubiquinone] iron-sulfur protein 2 mitochondrial; *EglN1*, Egl nine homolog 1; *STMP1*, short transmembrane protein 1; *PKM2*, pyruvate kinase M2 isoform; *HCM*, hypertrophic cardiomyopathy; *MAM*, mitochondria-associated endoplasmic reticulum membrane; *MDM*, mitochondrial diabetes mellitus; *MPTP*, mitochondrial-permeability transition pore; *m6 A*, N6 adenosine methylation: *OMM*, outer mitochondrial membrane; *PD*, Parkinson’s disease; *UCP2*, uncoupling protein 2; *SIRT3*, silent information regulator 3; and *AKAP1*, A-kinase anchor protein 1 mitochondrial

Disease name		Mechanism	Experimental model	reference
Cardiovascular diseases	Heart failure	SIRT3 Elevated mitochondrial acetylation	AC16 cells C57B/L6 mice	[28, 37]
		ROS increase Upregulation of NOX		[38]
	Cardiac ischemia–reperfusion injury	Ca2 + overload MPTP open Mitochondrial inner membrane permeabilization		[38]
	Diabetic cardiomyopathy	Oxidative damage to mitochondrial proteins and DNA	animal	[38]
	Myocardial infarct	Prx- 3 overexpression Interfere with mitochondrial oxidative stress Reduced hypertrophy, fibrosis and cardiomyocyte death	Prx- 3-transgenic mice	[86]
	Cardiac hypertrophy	Mitochondrial catalase overexpression	MCAT mice	[87]
	Accelerated age-associated cardiomyopathy	Mitochondrial polymerase γ mtDNA mutations and deletions	Mouse (mutator model)	[88]
	Neonatal mortality	MnSOD	Mouse	[89]
	Ischemic heart disease	MicroRNA- 210 Targeted glycerol- 3-phosphate dehydrogenase	MicroRNA- 210 deficient mice	[90]
	HCM	FARS2 deficiency	Fars2-knockdown zebrafish Fars2 gene changes mice	[91]
Diabetes	MDM	PGC- 1 α downregulation	Gene set enrichment analysis	[42]
	Diabetic cardiomyopathy	AGO2 decrease	Mouse models of diabetes and diabetes cardiomyopathy	[92]
		Bmal1 decrease Bcl2 transcription decrease Weakening the interaction between Bcl2/IP3R Increased release of Ca2 +	C57BL/6j mice	[93]
	DKD	AKAP1 Damage to mtDNA replication	Sprague–Dawley rats db/db mice	[45]
	Diabetic foot ulcer	METTL3 NDUFB5 m6 A modification	HUVECs cell Human samples C57BL/6 J mice	[94]
Nervous system disease	AD	Increase of C99 at the MAM Increase of ER-mitochondrial communication	Cell Human tissue	[51]
		Overexpression of β-amyloid OMM changes	APP/PS1 mouse	[53]
		Impaired mitophagy Accumulation of A β and Tau	Animal and cellular models of AD and in patients with sporadic late-onset AD	[95]
		Elevated mitochondrial Ca2 + levels	APP/PS1 Tg mouse	[52]
		PGC- 1α gene transfer Reduce β-secretase	APP23 mice	[96]
		m-AAA protease subunit AFG₃L₂ loss Mitochondrial transport defects Tau hyperphosphorylation	Mice	[97]
	PD	Mitochondrial complex I NDUFS2 lack	NDUFS2 knock out mice	[59]
		PIAS2-mediated blockade of IFN-β signaling	Cell Mice	[60]
		PINK mutation TOM complex Mitophagy	Cell	[61, 62]
	MELAS	m.3243 A > G mutation in MT-TL1 gene	Human plasma	[98, 99]
	Huntington’s disease	Mitochondria-targeting HSF1 mtDNA deletion	Mice	[100]
	Amyotrophic lateral sclerosis	DP- 43 triggers mtDNA Release	Cell Human tissue	[101]
	Leber’s hereditary optic neuropathy	Coenzyme Q10 m.3460 G > A mutation in mitochondrial protein ND1	Molecular dynamics and FEP simulations	[102]
Cancers	Ovarian cancer	Accumulation of lipid droplets Enhanced aerobic glycolysis	Cell	[76]
		Interaction between acylglycerol kinase and ribosomal protein L39	Cell Human tissue	[77]
		CRL4 CUL4 A/DDB1 Upregulating AMPKαThr172 and MFFSer172/Ser146 phosphorylation	Cell	[78]
		OTUB2 silencing Metabolic reprogramming	Human tissue	[69]
	lung cancer	AIF deficiency	Mice cell	[79]
		ATMLP elevation	Cell	[103]
		MARS2 MCU Attenuates mitochondrial Ca2 + influx	Cell	[104]
	Breast cancer	MYC, MCL1 Increase mtOXPHOS and the ROS	Human tissue Cell, mice	[85]
		Mitochondrial EglN1-AMPKα axis	cell	[105]
	Pancreatic cancer	UCP2 Drp1 phosphorylation	Mice	[82, 83]
	Liver cancer	Mitochondrial Micropeptide STMP1 Enhance mitochondrial fission	Human tissue Cell lines	[106]
	Glioma	PGC1α degradation Mitochondrial biogenesis	Human tissue Cell lines, mice	[107]
		HSP90-PKM2-Bcl2 axis	Cell lines	[108]

## Promising multiomics approaches in mitochondrial medicine

### Genomics

Since the genes for cytochrome c oxidase subunits I, II, and III, cytochrome b, 22 tRNAs, ATPase subunit 6 and the 12S and 16S rRNAs were identified in 1981 [12], researchers started to study mitochondrial proteins. Further, in 1986, the concept of genomics was firstly proposed to focus on the evolution, localization, structure, function and editing of genomes, as well as their impact on living organisms, the structural genomics, functional genomics, epigenomics, and metagenomics are also produced to better understand the causes and help with early detection and diagnosis [109, 110]. In 1988, the first human disease caused by mtDNA mutation was reported to find that Leber’s hereditary optic neuropathy was related to mtDNA mutation, with a conversion of arginine to histidine at codon 340 in the NADH dehydrogenase subunit 4 gene, and elimination of an Sfa NI site [111]. Mitochondrial diseases have become a common cause of genetic disorders. With the advancement of experimental technology, more and more potential mechanisms are being explored. It is difficult to diagnose the deficiency of multiple respiratory chain complex at molecular level. Genomics analysis plays important roles in this type of diseases. For example, (i) the whole exome sequencing (WES) analysis of 53 patients identified new mutations in four possible mitochondrial disease genes (PTCD1 (pentatricopeptide repeat-containing protein 1, mitochondrial), GARS (glycine–tRNA ligase), VARS2 (valine–tRNA ligase, mitochondrial), and FLAD1 (Flavin adenine dinucleotide synthase 1)), which provided a deeper understanding of mitochondrial defects [112]. (ii) Genome wide CRISPR Death Screen can identify genes necessary for OXPHOS and revealed a functional module (RPUSD3 (RNA pseudouridylate synthase domain-containing protein 3), FASTKD2 (FAST kinase domain-containing protein 2, mitochondrial), TRUB2 (pseudouridylate synthase TRUB2, mitochondrial), WBSCR16 (Williams-Beuren syndrome chromosomal region 16 protein), NGRN (neurite outgrowth-associated protein), and RPUSD4 (RNA pseudouridylate synthase domain-containing protein 4)), which can regulate intra mitochondrial translation and mitochondrial 16S rRNA [113]. (iii) The accumulation of mtDNA mutations leads to mitochondrial dysfunction, which is critical for glioblastoma multiforme (GBM), resulting in energy metabolism disorders and chemotherapy resistance [114]. The sequencing results of mitochondrial genomes from four GBM cell lines (M006x, M006xLo, M059 K, and M010b) revealed a new transition mutation (T-C transition in base pair 14,634) in hypoxia sensitive cell line M010b, which resulted in a single amino acid change in the gene encoding ND6 subunit of NADH ubiquinone oxidoreductase [115]. (iv) WES detected the mutation of SLC25 A4 that encodes mitochondrial adenosine diphosphate (ADP)/adenosine triphosphate (ATP) carrier 1 (AAC1), this mutation is well-recognized as the causes of mitochondrial diseases, such as childhood-onset mitochondrial myopathy, cardiomyopathy, adult-onset autosomal-dominant progressive external ophthalmoplegia, which can help people further study diseases and find suitable drug targets [116].

Moreover, genomics can identify the methylation of mtDNA, which is an important form of mtDNA modification and has clinical predictive value in tumors. DNA methylation research has shown that mutations in isocitrate dehydrogenase (IDH) are associated with glioma [117]. Under normal circumstances, IDH can convert isocitrate to 2-oxoglutarate (2-OG) in TCA cycle. When IDH mutates, it converts 2OG to a structural similar metabolite, (R)− 2-hydroxyglutarate ((R)− 2HG) [118], and can lead to high methylation of DNA and histones. Mutations of fumarate hydratase (FH), succinate dehydrogenase (SDH), and nicotinamide N-methyltransferase (NNMT) can also lead to methylation changes in cancer cells [119, 120]. Fumarate and succinate are inhibitors of multiple α-ketoglutarate-dependent dioxygenases, the accumulation of fumarate and succinate can be caused by mutations of FH and SDH in certain tumors, thus altering genome-wide histone methylation [120].

### Transcriptomics

Transcriptomics is to study gene expressions at the RNA level. The transcription of mtDNA has a unique mechanism, which is regulated by mitochondrial transcriptional complex that includes transcription factors POLRMT (mitochondrial RNA polymerase), TFB2M (mitochondrial transcription factor B2), and TFAM [121, 122], and known nuclear gene expression regulatory factors NRF- 1/2, PRC, PGC1-β, and PGC1-α. The gene expressions of the same cell are not completely same at its different growth stages and growth environments. Transcriptomics can provide the temporal and spatial regulatory patterns of genes, which is very helpful for understanding the dynamic processes of gene regulation. Four examples are taken here. (i) Sepsis-induced cardiomyopathy (SICM) is common in sepsis patients and is characterized by abnormal immune response. Due to cellular heterogeneity, it is difficult to understand the role of immune cell subsets in SICM [123]. By combining single-cell RNA sequencing with fate mapping in mouse model, the Mac1 subpopulation has distinct transcriptomic signatures and displays high expression of triggering receptor expressed on myeloid cells 2 (TREM2^++hi^). TREM2^hi^ Mac cells actively scavenge cardiomyocyte-ejected dysfunctional mitochondria, which suggest that it is a potential therapeutic target for SICM [123]. (ii) Transcriptomics is used to study the relationship between mitochondria and neurological diseases, which proposed two main novel mechanisms associated with neurological disorders [124]. (iii) Low concentrations of Mn can impair autophagy but do not interfere with the mitochondrial morphology and function of dopaminergic neuronal cells. Transcriptomics analysis of C57/BL6 mice that orally administered low-dose MnCl for 30 days revealed 508 upregulated DEGs and 509 downregulated DEGs. Pathway network analysis of these DEGs revealed autophagy and mitochondria-related pathways. Further study found that manganese treatment caused gene expression changes of multiple autophagy pathways, while mitochondrial dynamics and function-related genes were not significantly changed, Mn induced damage to autophagy and protein aggregation in dopaminergic neurons can be weakened by the inhibition of Drp1, and autophagy damage and mitochondrial dysfunction are two prominent features of neurodegenerative diseases, thus inhibiting Drp1 to improve mitochondrial function has become an attractive therapeutic target [124]. (iv) Whole transcriptome analysis revealed new mechanisms of subcutaneous adipose tissue (SAT) aging, which found that newly discovered glycogenin- 2 (GYG2) marker can be used to determine the age of SAT and is also a mitochondrial-related aging biomarker in human SAT [125].

Moreover, transcriptomics can identify RNA modifications such as N6 adenosine methylation (m6 A). The m6 A methylation is the most common RNA internal modification and participates in various physiological processes including DNA repair and lipid metabolism. The m6 A methylation is regulated by three types of protein regulators, including m6 A binding protein (reader), methyltransferase (writer), and demethylase (eraser) [126]. Once these m6 A methylation regulators occur m6a methylation, various diseases can arise, including tumors, neurological disorders, and more. m6 A can affect tumor occurrence and development by regulating the expression of various targets through post-transcriptional modifications, including leukemia, glioblastoma, ovarian cancer, and liver cancer [126–128]. m6 A methylation is common in cancer and is a potential biomarker and therapeutic target with potential clinical application value. For example, research has found that the demethylase FTO (alpha-ketoglutarate-dependent dioxygenase) can delay ovarian aging and may become a potential target for treating ovarian aging [129].

### Proteomics

Proteomics is to study a complete set of proteins expressed in a genome at a specific moment, which focuses on proteins as the research objective, including protein composition and its changes in cell, tissue, organism, protein expression level, PTM, and protein–protein interaction, to obtain a better understanding of disease etiology, cellular metabolism, and other processes at the level of protein. Mitochondrial protemics has important roles in clarification of phathophysiology of disease and biological life. For example, a study analyzed over 3300 mitochondria and mitochondria-related parts, which difined 901 high-confidence mitochondrial proteins [130] and further used subtractive proteomics and spatial proteomics to define the human high-confidence mitochondrial proteome that includes over 1100 mitochondrial proteins, of which 49 proteins have never been associated with mitochondria, and over 40% of mitochondrial proteins is associated with human diseases [131]. Moreover, multiple reversible protein PTMs are found to exist in the mitochondria, including phosphorylation, acetylation, and O-GlcNAcylation; and the number of known mitochondrial protein PTMs and target protein types has significantly increased with the development of proteomics technology, which demonstrates the prospects of mitochondria-targeted therapy [132]. Currently, MitoCarta has become the mitochondrial reference proteome for mammals, along with the mitochondrial protein composite index (IMPI) and mitochondrial high-confidence proteome [133]. This mitochondrial reference mapping aims to deepen our understanding of mitochondria, localize mitochondrial proteins, and identify PTMs to evaluate the regulation or disruption of mitochondria in diseases. Protein localization methods include split green fluorescent proteins (split-GFP), top-down proteomics, chemical crosslinking, and mitochondrially localized epitope tag [133]. It is a long-term task to establish a human mitochondrial protein atlas, which will further help understand mitochondrial PTMs for disease mechanism and drug development, provide a more accurate theoretical basis for exploring more effective treatment pathways, and continue to advance the medical development of mitochondrial therapy.

PTMomics is an important aspect of mitochondrial proteomcis, which studies different protein PTMs occurring at mitochondria. Some known PTMs such as ubiquitination, glycosylation, phosphorylation, methylation, and acetylation have been identified in mitochondria. Proteins with abnormal mitochondrial expression or modification can serve as biomarkers for disease prediction, prevention, and therapeutic targets. For example, (i) Drp1 in mitochondria has different PTMs, such as S-nitrosylation, SUMOylation, ubiquitination, and phosphorylation [134]; among them, phosphorylation at residues ser616, ser44, and ser637 in Drp1 regulates mitochondrial fission, and is associated with disease occurrence. Also, parkin can ubiquitinate Drp1 to affect proteasome degradation, which alters mitochondrial fusion-fission dynamics [21, 135]. (ii) Acetylation as a common PTM is divided into histone acetylation and non-histone acetylation, which exists in various parts of cells and affects most biological processes of cells. Histone acetylation occurs in the nucleus, promoting chromatin remodeling, transcription regulation, and DNA repair regulation. Non-histone acetylation participates in multiple biological processes, including gene transcription, protein folding, DNA damage repair, autophagy, cell signal transduction, metabolism, and cell division, as well as key cellular processes related to tumors [136]. Mitochondrial acetylation primarily refers to non-histone acetylation. Approximately 35% of mitochondrial proteins have at least one acetylation site [137]. Acetylation in the mitochondria is regulated by acetyltransferases and deacetylases. SIRT3 is an NAD^+^-dependent deacetylase in mitochondria, which regulates protein function and mitochondrial metabolism by removing acetyl groups from the mitochondrial proteins, and activate long-chain acyl CoA dehydrogenase (LCAD) to promote fatty acid oxidation [138], and superoxide dismutase 2 (SOD2) to enhance cellular antioxidant capacity [139]. In addition, excessive acetylation of mitochondrial proteins can accelerate glycolysis, thereby promoting the occurrence of malignant tumors. Because SIRT3 has deacetylation function to inhibit excessive acetylation of mitochondria, SIRT3 is currently a promising therapeutic target for various human diseases. Subsequent studies found that silent information regulator 4 (SIRT4) and silent information regulator 5 (SIRT5) also mediate deacetylation, but their deacetylation ability is weaker than that of SIRT3. Moreover, although relatively little research is conducted on acetyltransferases in mitochondria, previous studies have shown that some cytoplasmic and nuclear acetyltransferases (such as Gcn5) can enter the mitochondria under specific conditions and functions.

### Metabolomics

Metabolomics has two approaches—targeted metabolomics and non-targeted metabolomics, which can be used for individual detection and analysis, as well as combination with other omics analyses to reveal molecular mechanisms of complex disease processes, discover therapeutic targets, and establish biomarkers. Mitochondria are involved in energy metabolism associated with all diseases and life. Mitochondrial-related metabolomics can help better understand disease occurrence, and evaluate disease changes and treatment outcomes. Three examples are taken here: (i) serum metabolomics, as a highly sensitive and high-throughput feature, can discover potential biomarkers for colorectal cancer (CRC) [140, 141]. The ultra-performance liquid chromatography-mass spectrometry (UPLC-MS) analysis of serum metabolites of 61 healthy controls and 62 CRC patients revealed that CRC was associated with pantothenic acid, coenzyme A (CoA) biosynthesis, arginine biosynthesis, and pyrimidine metabolism; and after intervention, four specific serum metabolites (tyrosine (Tyr)-serine (Ser), N-phenylacetylasparticacid, tyrosyl-gamma-glutamate, and sphingosine) returned to a healthy level in CRC patients [140]. Another study used untargeted metabolomics to identify significant differential plasma metabolites in CRC patients relative to healthy controls, including amino acids, fatty acids, and nucleotides, further used targeted metabolomics to verify the difference of those metabolites (phenylalanine, tryptophan, creatinine, and other metabolites), and finally constructed a diagnostic model for CRC with high diagnostic accuracy and reproducibility [141]. (ii) The early diagnosis and treatment outcomes of sepsis are still unsatisfactory. Because circulating exosomes contain sepsis biomarkers and mediators, a study used UPLC-MS and ribonucleic acid sequencing (RNAseq) to analyze dynamic spectrum of serum exosomal proteins and RNAs in sepsis paptients, and then used LiquiChip assay, qRT PCR (quantitative reverse transcription polymerase chain reaction), and metabolomics to verify those results, which identified a total of 55 miRNAs, 354 proteins, 82 lncRNAs (long non-coding RNAs), and 195 mRNAs that were differentially expressed in serum exosomes of sepsis patients [142]. (iii) HCM is associated with metabolic disorders. A comprehensive multiomics analysis of HCM myocardial tissues revealed that multiple biochemical pathways were changed, including severe dysregulation of fatty acid metabolism, reduction of acylcarnitine, and accumulation of free fatty acids; ATP, ADP, and phosphocreatine were reduced in HCM heart; and mitochondrial genes involved in ATP synthesis and creatine kinase were reduced [143]; and also, electron microsopy found that mitochondrial damage was increased, and cristae density was decreased, which is consistent with decreased citrate synthase activity and mitochondrial oxidative respiration [143].

Application of multiomics in mitochondrial medicine is highlighted in Table 2.

**Table 2:** Application of multiomics in mitochondrial medicine. *AML*, acute myeloid leukemia; *SICM*, sepsis-induced cardiomyopathy; *POI*, premature ovarian insufficiency; *DEGs*, differentially expressed genes; *HCM*, hypertrophic cardiomyopathy; *IDH*, isocitrate dehydrogenase; *m6 A*, N6 adenosine methylation; *NNMT*, nicotinamide N-methyltransferase; *SOD2*, superoxide dismutase 2; *SIRT3*, silent information regulator 3; and *NADH*, nicotinamide adenine dinucleotide

Omics category	Disease or physiological process	Changes in omics	References
Genomics	Leber’s hereditary optic neuropathy	Arginine to a histidine at codon 340 in the NADH dehydrogenase subunit 4 gene eliminate Sfa NI site	[111]
	Mitochondrial respiratory chain complex deficiencies	VARS2, GARS, FLAD1, PTCD1	[112]
	OXPHOS	NGRN, WBSCR16, RPUSD3, RPUSD4, TRUB2, FASTKD2	[113]
	Glioblastoma	Mitochondrial complex I ND6 Subunit mutation D-LOOP T-C transition in base pair 14,634	[115]
	Mitochondrial disease	SLC25 A4 mutations	[116]
Transcriptomics	SICM	TREM2^hi^ Mac1 cells Maintain cardiomyocyte homeostasis	[123]
	Neurodegenerative diseases	Mn, Drp1	[124]
	Aging	GYG2, aging related biomarker	[125]
	OXPHOS	OXPHOS elevated Adjust cell division time promote B cell clonal expansion	[144]
	POI	119 mitochondrial DEGs	[145]
Proteomics	Cancer	NNMT Metabolic methylation Promote epigenetic remodeling	[119]
	Glioma	IDH DNA methylation	[118]
	Ovarian senescence	FTO Delay ovarian aging m6 A methylation	[146]
	Oxidative stress	SIRT3 SOD2 lysine residue deacetylation	[139]
	Neurodegenerative diseases	MAM S-palmitoylation	[147]
	Cancer	Protein lipoylation cell death	[148]
	AD	Mitochondrial proteins, APP and tau succinylation	[149]
Metabolomics	CRC	Arginine biosynthesis, pyrimidine metabolism, pantothenate, CoA biosynthesis	[140]
	CRC	Phenylalanine, tryptophan, creatinine	[141]
	Sepsis	Serum exosomes	[142]
	HCM	Dysregulation of fatty acid metabolism, reduction of acylcarnitine, and accumulation of free fatty acids	[143]
	Mitochondrial disease	Lactate, alanine, GDF- 15, α-hydroxybutyrate, N-lactoyl-amino acids, β-hydroxy acylcarnitines, β-hydroxy fatty acids	[99]
	Fatty liver disease	Resolvin D1, maresin 1	[150]

## Molecular alterations directly associated with mitochondrial impairments as attractive diagnostic and therapeutic targets

A series of mitochondrial dysfunction-related molecular pathway alterations have been found to directly associate with mitochondrial impairments, which are the attractive diagnositic and therapeutic targets. Several examples are taken as follows:

i. Immune-related molecular alterations: Pattern recognition receptors (PRRs) are expressed in immune and non-immune cells. PRRs can be activated by endogenous damage-associated molecular patterns (DAMPs) to usually trigger inflammation. Cellular stress and death affect the permeability of membranes to activate PRR through DAMP and its associated inflammatory responses. Mitochondria originated from α‐proteobacterium that is the ancestor of modern gram-negative bacteria [151]. Some of their components are extremely similar to those of bacterial molecules, which suggests that they may function as PRR ligands. Mitochondria are bi-membrane organelles, which together offer a dual layer of control segregating mitochondrial DAMPs (mtDAMPs) from their cognate PRRs. Injury makes mtDAMPs release into the circulation and induce immune responses [152]. mtDAMPs include formyl peptides and mtDNA, which activate human polymorphonuclear neutrophils through formyl peptide receptor- 1 and Toll-like receptor (TLR) 9, respectively. mtDAMPs promote PMN Ca^2+^ flux and phosphorylation of mitogen-activated protein kinases (MAPKs), thus leading to PMN migration and degranulation in vitro and in vivo.
ii. Regulatory cell death-related molecular alterations: Mitochondrion controls the apoptotic and necrotic forms of regulatory cell death, ultimately leading to irreversible mitochondrial permeability [151]. Among them, pro-apoptotic proteins Bax (Bcl- 2 associated X protein), and Bcl- 2 antagonist/killer factor 1 (BAK1) of the Bcl- 2 family related to mitochondria can cause changes in OMM permeability, promote the transport of cytochrome c between mitochondrial membrane gap and cytoplasm, cause the self-assembly of apoptotic complexes, and promote apoptosis. NLRP3 inflammasomes participate in the inflammatory response in various diseases, and mitochondria are involved in their activation of NLRP3 inflammasomes. When mtDNA is oxidized, it can bind to inflammasomes, linking cell apoptosis with the inflammatory response [153]. NLRP3 ubiquitination mediated by mitochondrial associated E3 ligase MARCH5 works in the activation of inflammasomes. MARCH5’s interaction with nucleoside triphosphatase domain of NLRP3 promotes polyubiquitination at residue lysine 27 (K27) connections on NLRP3’s residues lysine 324 (K324) and lysine 430 (K430), in order to form NEK7-NLRP3 oligomers, which then participate in the inflammatory response [154].
iii. Mitochondrial permeability-related molecular alterations: Mitochondria activate the cysteine protease family and caspase proteases to cause cell apoptosis, which is a programmed cell death that can eliminate aging cell and abnormal cell. Inner mitochondrial membrane permeability (MOMP) is a key factor that affects apoptosis. When cells are stimulated internally and externally, the IMM permeability changes to release cytochrome C and other proteins inside mitochondria, which can trigger the apoptotic signaling pathway, activate apoptosis-related proteins, such as the cysteine protease family (caspases), and ultimately lead to cell apoptosis.
iv. Cell apoptosis-related molecular alterations: There are usually two pathways for cell apoptosis, exogenous pathway and endogenous pathway. In the exogenous pathway, apoptosis is activated by activating caspase through extracellular signals. In the endogenous pathway, cell apoptosis is triggered by mitochondrial released apoptotic enzyme activating factor to activate caspase. These activated caspases can degrade important proteins within cells to cause apoptosis. Bcl- 2 family is crucial in endogenous pathways [155]. Of them, Bax is a pro apoptotic protein mainly located on the OMM, and Bcl- 2 is an anti-apoptotic protein mainly located on the OMM and endoplasmic reticulum. Bcl- 2 can inhibit the activity of Bax, and prevent changes in MOMP and the occurrence of apoptosis. Usually Bax is in an inactive state, while Bcl- 2 inhibits its activity by binding to Bax, thereby maintaining the survival of cells. When apoptosis signals occur in cells, the activity of Bcl- 2 protein may be activated by regulatory proteins such as Bad, leading to the release of Bax from Bcl- 2. Ultimately it causes changes in membrane potential, ROS, and the release of cytochrome C and AIF, to trigger the caspase cascade reaction and ultimately lead to cell apoptosis [155]. For example, when human bronchial epithelial cell line BEAS- 2B encounter industrial toxic cadmium, the MMP inside the cells decreases, ROS accumulates, Bcl- 2 expressions are downregulated, while Bax and caspase- 3 are upregulated. At the same time, the phosphorylation of P38, ERK (extracellular signal-regulated kinase), and JNK (c-Jun N-terminal kinase) were enhanced, and activate MAPK signaling pathway, ultimately leads to apoptosis of BEAS- 2B cells [156].
v. Inflammation-related molecular alterations: Mitochondrial dysfunction induced inflammation involves multiple signaling pathways, including inflammasome signaling pathway and cyclic GMP AMP synthase (cGAS) stimulator of interferon genes 1 (STING1) signaling pathway [151]. The cGAS STING1 signaling pathway involves the intracellular signaling of mtDNA-activated cGAS and STING1, which drives inflammation and is associated with caspase activation. In addition to serving as an effective cGAS agonist, mtDNA can also drive the activation of inflammasomes and the release of inflammatory factors [151]. Transforming growth factor-β (TGF-β) regulates cell differentiation, growth, and apoptosis, and its signal pathway induces autophagy in breast cancer, and HCC [157]. Research has found that TGF-β also induces autophagy in ovarian cancer, TGF-β can affect ovarian cancer cells by altering the TME. Overexpression of MARCH5 promotes TGFβ1-induced autophagy, which regulates TGF-β-SMAD2/3 pathway and the expressions of MARCH5 in cancer cells [24, 158]. The iTRAQ quantitative proteomics analysis of mitochondrial samples in ovarian tumors identified 1198 mtDEPs and 70 signaling pathways, and found metabolic pathways related to energy metabolism reprogramming in ovarian cancer [159].
vi. Mitophagy-related molecular alterations: Mitophagy is a mechanism that clears damaged mitochondria, and PINK1/Parkin signaling pathway works in it. Parkin, an E3 ubiquitin ligase, regulates mitophagy through various signals, such as PINK1/Parkin and LC3. Damaged mitochondria accumulate PINK1, recruit parkin, and label mitochondria for degradation [160]. In PD, PINK1 accumulates in damaged OMM, activates Parkin E3 ubiquitin ligase, recruits Parkin to dysfunctional mitochondria. Subsequently, Parkin ubiquitinates OMM proteins, and finally triggers selective autophagy [26, 161]. BNIP3/NIX also induces mitochondrial autophagy. When the ubiquitin independent mitochondrial autophagy receptor proteins BNIP and NIX were knocked out in Drp1 ineffective cell lines, it was confirmed that mitochondrial clearance depends on Drp1-dependent mitochondrial breakage and BNIP3/NIX-mediated mitochondrial autophagy [162].

Targeting mitochondrial dysfunction-related molecular pathways is exemplified in Table 3.

**Table 3:** Targeting mitochondrial dysfunction-related molecular pathways. *YAP*, yes-associated protein; *AMPK*, AMP-activated protein kinase; *4E-BPs*, 4E (eIF4E)-binding proteins; *NIX*, nip3 like protein X; *SOD2*, superoxide dismutase 2; *NAD*, nicotinamide adenine dinucleotide; *NADH*, nicotinamide adenine dinucleotide; *PI3 K*, phosphatidylinositol 3-kinase; *eNOS*, endothelial nitric oxide synthase; *GSK3*, glycogen synthase kinase 3; *AKT/PKB*, protein kinase B; *CTSB*, cathepsin B; *TEAD1*, TEA domain family member 1; *mTORC1*, mammalian target of rapamycin complex 1; and *4E-BPs*, eukaryotic translation initiation factor 4E (eIF4E)-binding proteins

Mitochondrial function	Signaling molecular pathways	References
Cell apoptosis	Bcl- 2/Bax YAP/Bcl- 2 Bcl- 2/Bax/cleaved caspase- 3 SIRT3/SOD2	[155, 156, 163, 164]
	PI3 K/Akt/Enos PI3 K/AKT/GSK3	[165, 166]
	Telomere–p53–PGC	[167]
Mitophagy	Mitophagy-ROS-CTSB-NLRP3	[168]
	Hippo signal YAP-TEAD1 inactivation	[169, 170]
	PINK1/parkin	[26, 160, 161]
	Wnt/β-catenin	[171, 172]
	LKB1-AMPK	[173–175]
	BNIP3/NIX	[162]
Immunoreaction	cGAS STING1	[151]
	NF-κB	[176, 177]
Mitochondrial biogenesis	PGC- 1α Sirt1/PGC- 1α	[54, 178, 179]
	mTORC1 4E-BPs	[180]
Mitochondria dynamics	cMAP	[181]
Oxidation reduction	NAD +/NADH	[182, 183]
	mito-HIF- 1α	[184]

## Supporting mitochondrial functionality and restoring compromised mitochondrial health—status quo and 3PM-guided future developments

### Natural compounds with therapeutic effects beneficial to the mitochondrial health

Both pre-clinical and clinical studies have shown that appropriate dietary supplementation with natural compounds, vitamins and cofactors effectively reduces mitochondrial stress, improving mitochondrial functions and protecting affected individuals against health-to-disease transition in primary care and against disease progression in secondary care. A comprehensive overview has been recently published by the dedicated working group of specialists in the field [3]. As emphasized by these experts, complementarity of the compounds used has to be carefully considered by specialists with multi-professional expertise, and treatment algorithms should be tailored to individualized patient profiles [3].

### Mitochondrial transplantation

Mitochondrial transplantation can improve disease symptoms and has the potential to treat various diseases [185, 186]. Mitochondrial transplantation can improve heart function and alleviate myocardial ischemia, and alleviate IRI. Clinical experiments had shown that mitochondrial transplantation has positive effects, providing more possibilities for heart transplantation [187–189]. Transplanting mitochondria derived from stem cells into damaged cells can improve cellular and muscle tissue function [190]. Nevertheless, several challenges persist in its clinical application, including successful isolation, quantity of isolated mitochondria, and preservation of their activity. Further technical advancements are necessary to address these issues [191]. Mitochondria from other sources can also help improve disease. Research has found that mitochondria from platelets can alleviate cognitive impairment related to diabetes in mice, which is expected to become a potential treatment for diabetes-associated cognitive impairment (DACI) [192]. Acetaminophen (APAP) is hepatotoxic and can damage the liver cells. Based on the APAP-induced liver injury, mitochondrial liver vein injection into rats showed that mitochondrial transplantation improved cell necrosis and inflammation. Compared with clinically applied N-acetylcysteine, the mitochondrial transplantation group had the lowest level of total oxidation. Therefore, further clinical research is need to be done in liver injury [193]. Mitochondrial transplantation has therapeutic potential for breast cancer. Transplanting mitochondria into breast cancer cells can inhibit the growth of it and reduce their drug resistance [194]. mtDNA activity can be restored by introducing mtDNA into damaged breast cancer cells through extracellular vesicles [195]. Mitochondrial transplantation also has great therapeutic potential in other cancer treatments, such as the therapeutic potential of astrocyte-derived mitochondrial transplantation for glioma [196].

### Mitochondrial gene editing

Efficient gene editing of mtDNA is a hot topic and challenge in the field. CRISPR-Cas9 system can edit the nuclear genome which is very efficient, it can also edit mtDNA. By fusing mitochondrial guide peptides upstream of Cas9 protein and configuring the 3′-end untranslated region (3′-UTR) sequence of mitochondrial genes downstream, a CRISPR/Cas9 system targeting mitochondria, mito-Cas9, was optimized and established. However, the editing technology of mtDNA is still immature and far from meeting the needs of clinical treatment.

A study has found that the CRISPR-free and RNA-free base editor DddA-derived cytosine base editor (DdCBE) developed through the bacterial cysteine deaminase toxin DddA can enable C • G-to-T • A to edit mtDNA. Gene editing can accurately modify mtDNA and alter mitochondrial OXPHOS [197]. By using transcription-activator-like effector-linked deaminases, basic group A in mtDNA can be converted to G with a success rate of 49% [198]. Transcription activator-like effector nuclease (TALEN) and Gene editing tools Zinc Finger Nuclease (ZFN) have also been used to edit the mitochondrial genome. TALEN and ZFN can induce DNA double-strand breaks and further modify genes, which are expected to provide more options for gene therapy of mitochondria-related diseases [199]. Another technique involving mitochondrial modification is mitochondrial replacement therapy, which includes prokaryotic spindle transfer, polar body transfer, and pronuclear transfer. This technology can modify the mitochondrial genes of embryos, separate nDNA from mtDNA, and avoid genetic diseases in offspring. However, there is still significant ethical controversy surrounding this technology [200].

### Clinically relevant illustrations of the 3PM-guided approach

Below we provide clinically relevant illustration by analysing individual patient cases.

*Patient case analysis 1: mitochondria-centered protection against health-to-disease transition—primary care.*

A female premenopausal patient, 38 years old with body mass index (BMI) = 19, generally healthy with a strong family predisposition to ischemic stroke. The patient is an evident Flammer syndrome phenotype (FSP) carrier demonstrating following characteristic patterns: cold hands and feet with a frequent feeling of being cold, low blood pressure, dizziness, shifted circadian rhythms with prolonged sleep onset, migraine with aura, altered reaction towards drugs, pronounced pain sensitivity, strong smell perception, specific psychosomatic patterns (pronounced meticulous personality), tinnitus, and slowed wound healing. The patient is concerned regarding her potential predisposition to ischemic stroke and chronic fatigue which she is suffering from. Indeed, per evidence, there is a correlation between FSP, ischemic-reperfusion episodes, increased apoptotic rates and compromised mitochondrial health [4, 5, 201–203]. Tear fluid analysis performed for the patient demonstrated a significantly increased autophagy level compared to the reference values in corresponding group of age reflecting well FSP-characteristic ischemic-reperfusion cellular damage (the know-how of 3PMedicon GmbH, Austria, performing internationally validated tests [204]), the methodology is described elsewhere [4]. Mitochondrial health relevant nutraceuticals were recommended to stabilize health condition of the patient [3].

*Patient case analysis 2: individualized rehabilitation program in secondary care.*

A female patient, 28 years old, BMI = 22 kg/m^2^, mother of two children, 2 years ago diagnosed with endometrium carcinoma, underwent a series of operations followed by chemotherapy; currently considered disease-free but suffers from chronic fatigue and depression. Accumulated research data demonstrate that cancer patients, who underwent an aggressive therapy, are strongly predisposed to ischemic stroke and mood disorders that per evidence are linked to compromised mitochondrial health. Contextually, an advanced 3PM approach in secondary care is strongly recommended to protect affected patients against secondary cascading pathologies [3, 205]. Tear fluid analysis performed for the patient demonstrated a significantly decreased mitophagy level compared to the reference values in corresponding group of age (the know-how of 3PMedicon GmbH, Austria, performing internationally validated tests [204]), the methodology is described elsewhere [4]. Mitochondrial health relevant nutraceuticals were recommended to stabilize health condition of the patient [3].

### Concluding remarks

**Fig. 2:**
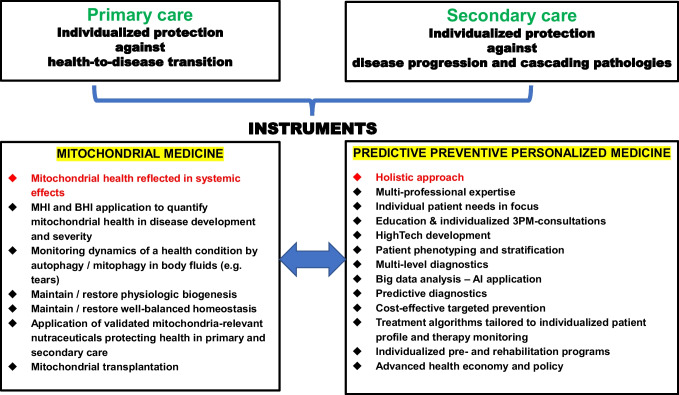
3PM-guided concepts of mitochondrial medicine [1–4, 7, 204–208]. MHI, mitochondrial health index; BHI, bioenergetic health index; AI, artificial intelligence; 3PM: predictive, preventive and personalized medicine

3PM-guided concepts of mitochondrial medicine are detailed in Fig. 2, which presents main pillars of the 3PM approaches and corresponding instruments of mitochondrial medicine. In particular as follows:

- Health risk assessment: The holistic approach is pivotal for an effective health risk assessment considering systemic effects analysed by individualized patient profiles in correlation with mitochondrial homeostasis in body fluids (such as the tear fluid); this is a powerful instrument for disease prediction (primary care) as well as for disease progression with consequently stabilized health condition monitoring (secondary care).
- Big data analysis and the AI application is essential for comprehensive patient phenotyping and stratification, multi-level diagnostics and treatment algorithms tailored to individualized patient profiles as well as innovative screening programs, amongst others; dynamics of mitochondrial health and homeostasis is an essential pillar of the in-depth analysis.
- Mitochondria-based preventive medicine linked to promoted 3PM literacy amongst healthcare givers and in the population (patient groups and their family members, regular check-up of healthy individuals, pre-pregnancy check-up, comprehensive medical services provided to top athletes, amongst others).
- Advanced healthcare economy and healthcare policy are anticipated to significantly benefit from the above listed 3PM strategies leading to reduced risks of modifiable (preventable) major pathologies in the population, increased treatments efficacy, highly motivated HighTech application in healthcare sector including consolidated multi-professional expertise, innovative diagnostics, targeted therapy application and drugs development.

## Data Availability

All the data used in this study are presented in this article.

## Code availability

All protein and gene accession codes are available in the Swiss-Prot and GenBank databases.

## Abbreviations

*AD*: Alzheimer’s disease
*ADOA*: Autosomal dominant optical atrophy
*AI*: Artificial intelligence
*AIF*: Apoptosis-inducing factor
*AML*: Acute myeloid leukemia
*AMPK*: AMP-activated protein kinase
*APAP*: Acetaminophen
*ATMLP*: AFAP1-AS1 translated mitochondrial-localized peptide
*BACE1*: β-APP cleaving enzyme 1
*BAK1*: Bcl- 2 antagonist/killer factor 1
*BHI*: Bioenergetic health index
*BMI*: Body mass index
*C99*: The amyloid precursor protein 99-aa C-terminal fragment
*CA9*: Carbonic anhydrase 9
*CDK5*: Cyclin dependent kinase 5
*cGAS*: Cyclic GMP AMP synthase
*COXI*: Cytochrome c oxidase subunit 1
*CRC*: Colorectal cancer
*CRISPR*: Clustered regularly interspaced short palindromic repeats
*DAMPs*: Damage-associated molecular patterns
*DEGs*: Differentially expressed genes
*DKD*: Diabetes kidney disease
*Drp1*: Dynamin-related protein 1
*FARS2*: The mitochondrial phenylalanyl-tRNA synthetase
*4E-BPs*: 4E (eIF4E)-binding proteins
*FH*: Fumarate hydratase
*Fis1*: Mitochondrial fission protein 1
*FSP*: Flammer syndrome phenotype
*FUNDC1*: FUN14 domain containing 1
*HCC*: Hepatocellular carcinoma
*HCM*: Hypertrophic cardiomyopathy
*HGSOC*: High-grade serous ovarian cancer
*HUVECs*: Human umbilical vein endothelial cells
*ICI*: Immune checkpoint inhibitor
*IDH*: Isocitrate dehydrogenase
*IMM*: Inner mitochondrial membrane
*IMPI*: Mitochondrial protein composite index
*IRI*: Ischemia-reperfusion injury
*iTRAQ*: Isobaric tags for relative and absolute quantitation
*KDM3 A*: Lysine demethylase 3 A
*Larp1*: La-related protein 1
*LCAD*: Long-chain acyl CoA dehydrogenase
*MAM*: Mitochondria-associated endoplasmic reticulum membrane
*MAPKs*: Mitogen-activated protein kinases
*MARCH5*: Membrane-associated RING-CH 5
*MARS2*: Mitochondrial methionyl-tRNA synthetase
*MCU*: Mitochondrial calcium uniporter
*MDM*: Mitochondrial diabetes mellitus
*MELAS*: Mitochondrial encephalomyopathy, lactic acidosis, and stroke-like episodes
*Mfn1*: Mitofusin 1
*Mfn2*: Mitofusin 2
*MHI*: Mitochondrial health index
*MIEF2*: Mitochondrial elongation factor 2
*MMP*: Mitochondrial membrane potential
*MnSOD*: Mn-superoxide dismutase
*MOMP*: Mitochondrial membrane permeability
*MPP*: Mitochondrial processing peptidases
*MPTP*: Mitochondrial-permeability transition pore
*mtDEPs*: Mitochondrial differentially expressed proteins
*mtDNA*: Mitochondrial DNA
*mtHSP70*: Mitochondrial heat-shock protein 70
*m6 A*: N6 adenosine methylation
*NAD*: Nicotinamide adenine dinucleotide
*ND5*: NADH-ubiquinone oxidoreductase chain 5
*nDNA*: Nuclear DNA
*NDUFB5*: Ubiquinone oxidoreductase subunit B5
*ND5*: NADH-ubiquinone oxidoreductase chain 1
*NIX*: Nip3 like protein X
*NLRP3*: NOD-like receptor with pyrin domain 3
*NNMT*: Nicotinamide N-methyltransferase
*NRF1*: Nuclear respiratory factor 1
*NRF2*: Nuclear respiratory factor 2
*OMM*: Outer mitochondrial membrane
*Opa1*: Optic atrophy 1
*OTUB2*: Otubain 2
*PD*: Parkinson’s disease
*POI*: Premature ovarian insufficiency
*PIAS2*: Protein inhibitor of activated STAT 2
*PPAR*: Peroxisome proliferator-activated receptor
*PPPM*: Predictive, preventive and personalized medicine
*PRRs*: Pattern recognition receptors
*PTM*: Posttranslational modification
*SAM*: Sorting and assembly machinery
*SAT*: Subcutaneous adipose tissue
*SDH*: Succinate dehydrogenase
*SICM*: Sepsis-induced cardiomyopathy
*SIRT1*: Silent information regulator 1
*SIRT3*: Silent information regulator 3
*SIRT4*: Silent information regulator 4
*SIRT5*: Silent information regulator 5
*SOD2*: Superoxide dismutase 2
*STING1*: Stimulator of interferon genes 1
*TALEN*: Transcription activator-like effector nuclease
*Tau*: Tubulin associated unit
*TFAM*: Transcription factor A mitochondrial
*TGF-β*: Transforming growth factor-β
*TLR*: Toll-like receptor
*TME*: Tumor microenvironment
*TNBC*: Triple negative breast cancer
*TOM*: Outer membrane transferase
*TREM2*: Triggering receptor expressed on myeloid cells 2
*T2D*: Type 2 diabetes
*3*′-*UTR*: 3′-End untranslated region
*UCP2*: Uncoupling protein 2
*WES*: Whole exome sequencing
*YAP*: Yes-associated protein
*ZFN*: Zinc finger nuclease
